# The impact of climate change on the medical profession – a newly implemented course on medical ecology

**DOI:** 10.3205/zma001612

**Published:** 2023-05-15

**Authors:** Claudia Gundacker, Monika Himmelbauer

**Affiliations:** 1 Medical University of Vienna, Institute of Medical Genetics, Vienna, Austria; 2 Medical University of Vienna, Teaching Center, Vienna, Austria

**Keywords:** medical ecology, climate change, multidimensional learning

## Abstract

**Objective:**

The consequences of climate change on health care systems as well as the individual involvement in climate change has not been a focus of the study of human medicine. Therefore, the lecture and practical course medical ecology have been reorganized to reflect the increasing importance of this topic. In order to be available to all students, this course was included in the core curriculum of the first year of study in human medicine.

**Methodology:**

The teaching concept is based on the method “multidimensional learning”. The theoretical examination of environmental changes, especially climate change, is placed at the starting point within the framework of a lecture, followed by the translation of theoretical principles into practical knowledge by calculating the ecological footprint and subsequent reflection on the newly learned content. The project was evaluated by means of a self-constructed course evaluation instrument (three feedback questions) and an internal university online tool.

**Results:**

656 students (100%) described the most important knowledge they gained in the course. One third of the students (N=218) indicate that they would like to participate in a more advanced seminar. 137 students comment on specific aspects. Overall, students express great interest in the topic of medical ecology. They reflect in a remarkably (self-)critical way on the individual contribution to climate change and can clearly name the health consequences of climate change. The contents should be expanded in a more in-depth seminar.

**Conclusion:**

The concept of the course has proven to be purposeful in order to prepare relevant and complex contents of medical ecology in an understandable way. Both lecture and practical course should be further developed accordingly.

## Zusammenfassung

**Zielsetzung:**

Die Folgen des Klimawandels auf die Gesundheitssysteme, als auch die individuelle Mitbeteiligung am Klimawandel standen bislang nicht im Fokus des Studiums Humanmedizin. Der zunehmenden Bedeutung des Themas Rechnung tragend, wurden Vorlesung und Praktikum zur medizinischen Ökologie deshalb neu ausgerichtet. Um alle Studierenden zu erreichen, wurde diese Lehrveranstaltung im Kerncurriculum des ersten Studienjahres Humanmedizin verankert.

**Methodik:**

Das Lehrkonzept basiert auf der Lernmethode „mehrdimensionales Lernen“. Die theoretische Auseinandersetzung mit Umweltveränderungen, insbesondere dem Klimawandel, wird im Rahmen einer Vorlesung an den Ausgangspunkt gestellt, gefolgt von der Umsetzung der theoretischen Grundlagen in praktisches Wissen durch Berechnung des ökologischen Fußabdrucks und daran anschließender Reflexion zu den neu gelernten Inhalten. Das Projekt wurde mittels eines selbst konstruierten Kursbewertungsinstruments (drei Feedback-Fragen) sowie eines universitätsinternen Online-Tools evaluiert.

**Ergebnisse:**

656 Studierende (100%) beschreiben die wichtigsten Erkenntnisse, die sie in der Lehrveranstaltung gewonnen haben. Ein Drittel der Studierenden (N=218) gibt an, an einem weiterführenden Seminar teilnehmen zu wollen. 137 Studierende kommentieren spezifische Aspekte. Insgesamt bekunden die Studierenden großes Interesse für das Thema Medizinische Ökologie. Sie reflektieren in bemerkenswert (selbst)kritischer Weise den individuellen Anteil am Klimawandel und können die Gesundheitsfolgen des Klimawandels klar benennen. Die Inhalte sollen in einem vertiefenden Seminar ausgebaut werden.

**Schlussfolgerung:**

Das Konzept der Lehrveranstaltung hat sich als zielführend erwiesen, um relevante und komplexe Inhalte der Medizinischen Ökologie verständlich aufzubereiten. Sowohl Vorlesung als auch Praktikum sollen entsprechend weiterentwickelt werden.

## 1. Introduction

### 1.1. Description of the subject area

Medical ecology is concerned with anthropogenic changes in the environment and their repercussions on human health. These include natural-resource use (overuse of soils, waters, natural landscapes), environmental pollution (industrial chemicals, environmental pollutants, noise, traffic emissions, high-energy radiation, light pollution), changes in biodiversity (spread of species, reduction of biodiversity), genetic engineering (genetically modified species), and global warming (climate change due to globally increasing greenhouse gas emissions). 

In 2012, 12.6 million deaths (23% of all deaths worldwide) were attributed to modifiable environmental factors, many of which are related to climate change [1]. The latest report from the Intergovernmental Panel on Climate Change (IPCC) [2] demonstrates that many climate impacts, including extreme events, have become more intense and frequent and that these will persist in the coming decades, accompanied by continued increases in temperatures. In all emissions scenarios examined, global mean surface temperature will reach +1.5°C relative to pre-industrial levels in the early 2030s, rising to +1.6°C to +2.4°C by 2050.

The challenge for global health systems is to be prepared for heat waves, changing work capacity, extreme weather events (wildfires, floods, drought), climate-related infectious diseases, malnutrition, migration, and related illnesses and deaths [3]. 

However, the “climate change crisis” is also seen as an opportunity to ensure global health in the 21^st^ century [4]. A central prerequisite is to inform future physicians about the expected consequences for the medical profession and to create awareness among medical students about the anthropogenic contribution to climate change.

#### 1.2. Motivation and goals

The central motivation was to train students to recognize connections between anthropogenic global environmental change and associated local health impacts. Heat waves associated with climate change and their consequences are relatively simple to understand. Health consequences based on the complex interplay of global warming and other environmental changes require didactic preparation in a compact form. Medical students predominantly need a basic understanding that humans are part of the environment and should act accordingly in a precautionary manner. Another motivation was to create awareness that changes in ecosystems and their biomes have a lasting effect, with medium- and long-term consequences for human life. The newly implemented course is thus intended to offer a presentation of the crucial facts under the premise of “information instead of uncertainty”.

## 2. Project description

### 2.1. Significance and focus of the course

**Figure 1:**
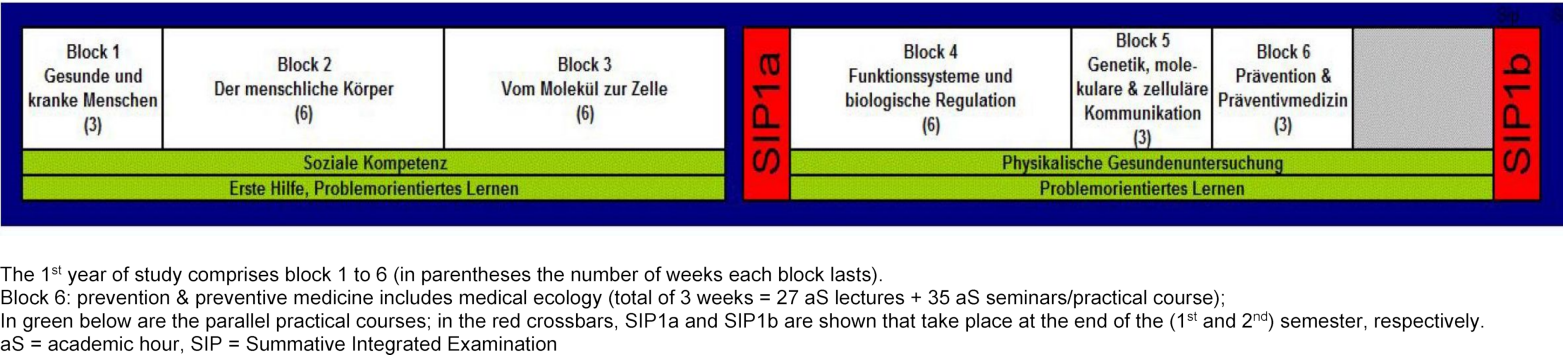
Curriculum Human Medicine 2020/21 (1^st^ academic year), german version

Since 2003, medical ecology has been taught at the Medical University of Vienna as part of the modular (i.e. in blocks) study program in Human Medicine in Block 6 of the first year (see figure 1 (Fig. 1)). Although anthropogenic environmental change (including the anthropogenic greenhouse effect) was taught until 2020, explicit impacts on health care systems and individual contribution to climate change were not a focus. Taking into account the increasing importance of the topic, the lecture and practical course medical ecology were realigned in the academic year 2020/21, i.e. previously taught parasitology is now taught separately in a lecture, and with regard to environmental changes, the focus was on the two major topics climate change and biodiversity.

#### 2.2. Design and structuring of the course 

In the study of human medicine, about 650 students per year are collectively educated in lectures. The practical courses are held in small groups of 10 participants – if teaching in physical presence is possible – in order to enable an intensive exchange as well as interaction.

The course Medical Ecology consists of a lecture and a practical course. The lecture Introduction to Medical Ecology (1 academic hour = aS) gives an overview of global warming (causes, predictions, health impacts) and biodiversity (loss of habitat and species, alien species, health impacts). 

The subsequent medical ecology practicum includes two teaching units (2 aS).

In teaching unit 1 (1 aS, self-study), students calculate their individual ecological footprint and transfer the values in global hectar to housing, nutrition, mobility and consumption as well as the overall result and the ratio (%) to the average footprint in Austria into an Excel spreadsheet on the Moodle teaching platform. In addition, knowledge about environmental changes and health impacts is tested by multiple choice (MC) questions and free text answers. In the second teaching unit (1 aS, after transmission of the group result via Moodle), the students analyze and reflect in small groups the individual result and group result in relation to the footprint in Austria and the global footprint, respectively. 

#### 2.3. Learning objectives

The learning objectives of the course refer to the acquisition of technical cognitive skills, but also to affective/attitudinal competencies and were elaborated by the course instructor in consultation with the block planning team and the curriculum commission for human medicine. Sample learning objectives for the course are listed in table 1 (Tab. 1). 

The learning objectives of the practical course, which has an immanent examination character, have been achieved if 75% of the tasks in the seminar have been fulfilled (calculation of the ecological footprint, self-study) or 75% of the examination questions (10 multiple choice (MC) questions and 8 free text questions) have been answered correctly. The lecture content was also examined as part of the Summative Integrated Examination at the end of the first year of study (SIP 1).

#### 2.4. Didactic approach

The teaching concept is based on the learning method “multidimensional learning”. This describes in didactics the extension and expansion of learning to several areas of the ability spectrum. The “multidimensional learning” aims at a structural change of the learning processes, in order to meet the different learning prerequisites on the one hand, on the other hand the different factual aspects of the learning material in the best possible way [5]. In our project, the theoretical discussion of environmental changes, especially climate change, in the context of a lecture is the starting point, followed by the implementation of practical knowledge by calculating the ecological footprint and subsequent reflection on the newly learned content. In addition, feedback (meta-communication) on the course is to be given afterwards.

Due to the COVID-19 pandemic, the course, which took place for the first time in SS 2021, was conducted in a distant learning format. This was easier to implement with regard to the synchronously held lecture than with regard to the practical course. The opportunity for in-depth discussion was intended in particular for the practical course, but could only be realized to a limited extent in the distant learning format.

#### 2.5. Evaluation and quality assurance

The project was evaluated in terms of quality assurance by means of a self-constructed course evaluation instrument (three feedback questions) (1), as well as additionally assessed by the university-internal online evaluation (2). Both surveys were not mandatory and were conducted (1) after the end of the seminar and (2) shortly after the end of Block 6 (at the end of SS 2021).

##### In-course survey

Since this was the first time the course was conducted, final reflection and feedback was requested. The goal was to determine the subjective perception of the students on the topic of climate change and to clarify whether there is a need for a more in-depth seminar in order to optimize the course in the future. Two open and one closed question were used, which were subsequently analyzed descriptively and qualitatively.

*In your small group, form five groups of two and discuss the following points, then please write them down in key words.*

*Question 1: What were the most important insights you gained in the Medical Ecology courses? (Free text)*

*Question 2: Would you want to attend an in-depth seminar on this topic?*

□* Yes *

□* No*

*Question 3: If you have any comments or suggestions, please note them here: (free text).*

According to the qualitatively oriented approach, the evaluation aspects of the open questions were generated close to or from the material [6]. In doing so, an inductive category development was carried out, which was oriented towards systematic reduction processes. The following categories could be distilled out: High general relevance of the topic and interest in the course, lack of clinical relevance, problems of the distant learning format.

##### University internal online evaluation 

All students who had completed the medical ecology course were asked by the university’s internal evaluation and quality assurance office to complete an evaluation form in digital form. To ensure privacy, the survey was voluntary and anonymous. The questions were related to the curriculum element (three general questions), the lecture (7 questions), and the practicum (12 questions). The evaluation of the questions was presented on a 4-point Likert scale (1=strongly disagree, 2=somewhat disagree, 3=somewhat agree, 4=strongly agree, respectively, 1=very poor, 2=rather poor, 3=rather good, 4=very good). The descriptive analysis of the online evaluation using IBM SPSS was performed by the staff office.

## 3. Results

### 3.1. Student collective 

656 students (350 females, 276 males, 3 diverse, 27 no response) participated in the course with a mean age of 21.4±2.7 years. All participants completed the course positively. There was feedback from all 66 small groups. The response of reflection and feedback was 100% for question 1 (most important findings from the medical ecology course) and 98% for question 2 (participation in in-depth seminar). 21% of students provided additional comments and suggestions (question 3). 

#### 3.2. Course-internal survey (reflection and feedback of the students)

##### 3.2.1. The most important insights gained

The qualitative evaluation indicates that there is a great interest in this topic, that some students were concerned, that curiosity and surprise were raised, and awareness created that environmental changes have an impact on the medical profession (for free text examples see table 2 (Tab. 2)).

##### 3.2.2. In-depth seminar

One third of the students (N=218) state that they would like to participate in an in-depth seminar, 422 students (64%) do not want to. 16 students do not indicate this.

##### 3.2.3. Feedback of the students on the course

Comments from 137 students (see table 3 (Tab. 3) for free text samples) who provided additional feedback can be qualitatively divided into four categories: High general relevance of the topic, high interest in the course, lack of clinical relevance, problems with the distant learning format.

77 students rated the course as important and interesting. Especially the calculation of the ecological footprint was perceived as enriching. Another 25 students also attribute high importance and relevance to the course, but miss the clinical relevance. 

35 students dedicated their comments to the design of the course, criticizing the distance learning format or missing the opportunity for discussion.

#### 3.3. University internal online evaluation

118 students (18%) participated in the online evaluation. Of these, 75% state that the learning content presented in the course is relevant to their professional career (as a physician or medical scientist), with 26% strongly agreeing and 49% somewhat agreeing. 71% state that the curriculum element requirements placed on them were spot on. The response rate for the online evaluation appears low, but is not unusual for online surveys [7].

## 4. Discussion

### 4.1. Achievement of learning objectives

All students fulfilled the assignment (calculation of the ecological footprint in the context of one academic hour of self-study) and achieved the learning objectives, i.e. 75% of the examination questions (10 MC and 8 free text questions) were answered correctly. The qualitative evaluation of the open-ended questions indicates that the students reflected in a remarkably (self)critical way on their individual impact on climate change and were able to clearly name the health consequences of climate change. Thus, the central content of medical ecology (How do we change our environment and how does that affect our health?) was adequately covered in an extremely relevant area. 

The results also demonstrate that the focused teaching on the health impacts of climate change generated great interest among the students. It should be mentioned here that both the lecture and practical course additionally cover other environmental changes^1^ whose effects are not immediately noticeable but which may become relevant in the future.

The anchoring of medical ecology (in the narrower sense: climate protection is health protection), is not or only rarely the case in the curricula of human medicine, sometimes as a postgraduate training [https://iph.charite.de/en/research/climate_change_and_health/]. As Planetary Health, it is more at home in Public Health curricula [8], [https://www.klimawandel-gesundheit.de/]. Overall, however, the importance of the topic to human medicine is recognized [9], [10], [11].

Student feedback also underscores the need to teach medical ecology in the regular curriculum, but also the need for more in-depth instruction on climate change impacts. The quantitative as well as qualitative evaluation of the feedback questions proves that the topic is considered important or extremely important. However, although for about two thirds of the students (64%) the content is sufficiently covered, one third of the students (218 out of 656) would like to have a more in-depth seminar. This wish is generally aimed at more differentiated content, both in terms of practice and actual application of knowledge on the topic of climate medicine (e.g. vaccinations), as well as the involvement of experts on specific topics. The low response rate of the online evaluation could be a limitation regarding the interpretation of the quantitative results. However, the agreement between these and the qualitative analyses indicates that the results are representative.

#### 4.2. Didactics

The didactic concept “multidimensional learning” (linking theoretical content with the implementation in practical knowledge by calculating the ecological footprint and subsequent reflection on newly learned content) was well received by the students and could also be implemented – as can be seen from the feedback on the most important insights gained and the reflection on the course as a whole.

The course took place for the first time in SS 2021. The short-term changeover to the distant learning format – especially for the practical course – presented a challenge. Thus, an in-depth discussion of the contents in the Distant Learning format could hardly be realized. 

The possibility to communicate with the students via Moodle was used for correcting serious mistakes. In addition, we used the Moodle tool as an opportunity to send a feedback letter to the students to specifically address the most common errors.

## 5. Conclusion

The concept of the course proved to be purposeful in presenting relevant and complex content of medical ecology in an understandable way. Both lecture and practical course should be further developed accordingly, both in terms of improving teaching quality and cross-linking in the curriculum, e.g. reference should be made to infectious diseases that will be lectured in subsequent years. 

Due to high demand, an in-depth seminar will be offered starting in WS 2022/23. In this seminar, students will have the opportunity to lecture on specific topics as well as to discuss with experts. For the future, it would make sense to integrate further courses on Planetary Health into the curriculum of human medicine in order to sensitize students to this topic and to expand their knowledge profoundly in the sense of a learning spiral.

## Notes

^1^ Alien species (neobiota) as disease vectors; loss of biodiversity (loss of habitat, loss of species as reservoirs for bioactive substances that could be used as drugs); genetically modified organisms.

## Acknowledgements

We wish to thank Sebastian Granitzer, Raimund Widhalm and Martin Forsthuber for their support in the development of the practical course and Tanja Paulmichl for her assistance in the evaluation of the feedback forms. We sincerely thank Prof. Harald Sitte for the discussion on the connectivity of the course content to other parts of the curriculum.

## Competing interests

The authors declare that they have no competing interests. 

## Figures and Tables

**Table 1:**
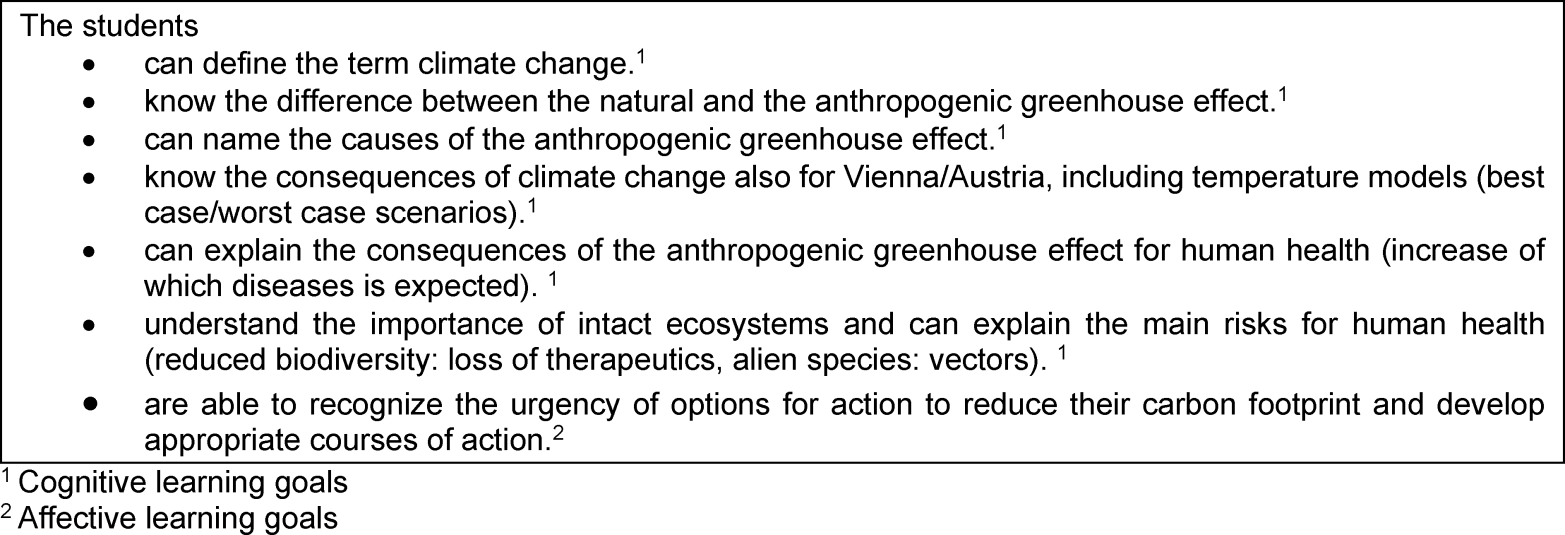
Learning objectives medical ecology

**Table 2:**
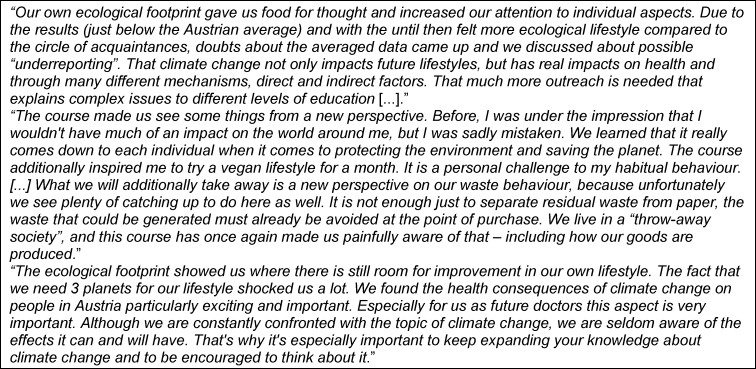
Examples of the most important insights gained

**Table 3:**
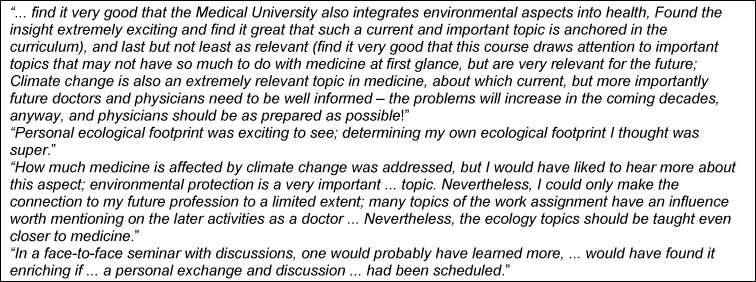
Examples of student feedback on the course
